# Factors associated with postpartum initiation of anti-hypertensive medication after hospital discharge among individuals with hypertensive disorders of pregnancy in a remote monitoring program

**DOI:** 10.21203/rs.3.rs-2761676/v1

**Published:** 2023-04-03

**Authors:** Alisse Hauspurg, Kripa Venkatakrishnan, Latima Collins, Malamo Countouris, Jacob Larkin, Beth Quinn, Nuzhat Kabir, Lara Lemon, Hyagriv Simhan

**Affiliations:** University of Pittsburgh; University of Pittsburgh

**Keywords:** hypertension, pregnancy, remote monitoring

## Abstract

**Importance:**

Following a hypertensive disorder of pregnancy, hypertension can worsen in the postpartum period following hospital discharge. Risk factors for hypertension exacerbation and associated outcomes have not been well characterized.

**Objective:**

We sought to identify risk factors and characterize outcomes for individuals requiring initiation of anti-hypertensive medication following hospital discharge postpartum through our hospital system’s remote blood pressure management program.

**Design:**

We performed a cohort study of individuals delivered between 9/2019–6/2021 and enrolled in our remote blood pressure monitoring program, which utilizes standardized protocols for anti-hypertensive medication initiation postpartum.

**Setting:**

Postpartum unit at a referral hospital

**Participants:**

Population-based sample of individuals with a hypertensive disorder of pregnancy (HDP, preeclampsia or gestational hypertension) and no pre-pregnancy hypertension.

**Exposure:**

Anti-hypertensive medication initiation timing: no anti-hypertensive medications, initiation prior to hospital discharge postpartum, and initiation after hospital discharge postpartum

**Main outcomes:**

Postpartum readmission and emergency room visits

**Results:**

Of 2,705 individuals in our cohort, 1,458 (54%) required no anti-hypertensive medications postpartum, 637 individuals (24%) were discharged on anti-hypertensive medications, and 610 (23%) required initiation of anti-hypertensive agents after discharge. Utilizing an inpatient threshold of ≥ 150/100 mmHg in line with current obstetric guidelines for medication initiation postpartum fails to identify 385 (63%) of individuals who required medication initiation after discharge. These individuals had higher home blood pressures, increased odds of Emergency Room visits [aOR 2.22 (95%CI 1.65–2.98)] and hospital readmissions postpartum [aOR 5.73 (95%CI 3.72–8.82)] compared with individuals discharged on no medications.

**Conclusions and Relevance:**

Over 20% of individuals with hypertensive disorders of pregnancy required initiation of anti-hypertensive medications after hospital discharge. Current blood pressure guidelines for medication initiation fail to identify the majority of these individuals during delivery hospitalization. These data support the critical role of remote blood pressure monitoring programs and highlight the need for improved tools for risk strati cation and liberalization of thresholds for medication initiation postpartum.

## Introduction

Maternal morbidity and mortality are increasing at an alarming rate in the United States, the majority of which occurs in the postpartum period.^1^ Hypertension complicates up to 20% of pregnancies in the United States and is a Significant contributor to maternal morbidity and mortality in the postpartum period.^1–3^ Despite the fact that hypertension is the most common reason for postpartum hospital readmission and a major contributor to maternal morbidity, prior studies and clinical management guidelines focus primarily on antepartum and intrapartum management, with little emphasis placed on postpartum management or blood pressure thresholds for anti-hypertensive initiation.^4^

The American College of Obstetricians and Gynecologists (ACOG) recommends a single blood pressure check between 3–10 days postpartum for individuals with a hypertensive disorder of pregnancy.^4,5^ Those who have ongoing or uncontrolled hypertension needing medication titration are typically seen more frequently in the postpartum period for medication management. There are no clear guidelines on optimal blood pressure management in the postpartum period, resulting in variation by provider and institution.^6^ ACOG suggests that treatment be initiated for persistent blood pressure ≥ 150/100 mmHg; however, these recommendations do not address the expected exacerbation of hypertension that is seen at days 4–7 postpartum.^7,8^ Severe hypertension (≥ 160/110mmHg) is associated with a higher risk of severe maternal morbidity postpartum, particularly stroke, heart failure, and seizure.^9,10^

Our objectives were to, first, identify risk factors for initiation of anti-hypertensive medication following hospital discharge postpartum; second, to characterize associated outcomes for individuals initiated on anti-hypertensive medications following hospital discharge and, third, to interrogate the performance of various blood pressure thresholds for identifying inpatient individuals ultimately requiring anti-hypertensive medications within a remote blood pressure monitoring program.

## Methods

### Cohort

This study is part an ongoing quality improvement project that included postpartum individuals admitted to the postpartum unit of a hospital system (University of Pittsburgh Medical Center, UPMC Magee-Womens Hospital, UPMC Northwest and UPMC Horizon) between September 2019 and June 2021. Eligible individuals have one of the following hypertension-related diagnoses: gestational hypertension, preeclampsia, eclampsia, or new onset postpartum hypertension. As our focus was on new-onset hypertensive disorders of pregnancy, postpartum individuals with pre-pregnancy chronic hypertension were excluded from this analysis. To be included in the program, individuals must be English-speaking and have access to a text messaging-enabled mobile device. Diagnoses of HDP are made by the clinical care team according to ACOG criteria.^4^ Regardless of diagnosis, hypertension in pregnancy is defined as blood pressure ≥ 140 mmHg systolic or 90 mmHg diastolic. Maternal, obstetrical and sociodemographic data are obtained from the electronic medical record and subsequently the Clinical Data Warehouse (CDW) at UPMC. The program was approved by the University of Pittsburgh Medical Center Quality Improvement Review Committee and the University of Pittsburgh Institutional Review Board. The data collection and analysis was approved as an exempt study posing no greater than minimal risk, thus written informed consent was not required. The reporting followed the Strengthening the Reporting of Observational Studies in Epidemiology (STROBE) reporting guidelines.

### Remote monitoring program

A description of the remote monitoring program has been previously described.^11^ Briefly, we created a remote patient monitoring platform that leveraged Vivify Health^®^ as its vendor. The monitoring platform is integrated with electronic health records and the CDW for both ordering and results. Individuals are enrolled in the program by their primary obstetric provider while inpatient on the postpartum unit or after a readmission postpartum. For this analysis, we included only individuals enrolled during the delivery admission. After identification and verification of eligibility, the provider places an order in the electronic medical record with the patient’s phone number which automatically generates a text message to enroll the patient. We utilize the A&D UA-651 (A&D Medical; San Jose, California) automatic upper arm blood pressure monitor. The patient is trained on use of the blood pressure device by a program-specific nurse educator prior to discharge from the hospital. During program enrollment, bedside nursing staff record blood pressure on both the home blood pressure monitoring device and the hospital device to confirm accuracy. The program is managed through a nurse-staffed UPMC call center with documentation of calls and blood pressures directly into the electronic medical record.

We designed nursing call center-driven blood pressure management and treatment algorithms that were developed by local expert stakeholders, consistent with national guidelines on goals for hypertension management postpartum.^12^ Following discharge from the hospital, individuals are prompted to check their blood pressure at least daily for the first two weeks of the program and between 3–5 times per week for the remainder of the program through six weeks postpartum. Blood pressures are reported utilizing text messaging. The initial choice of anti-hypertensive agent is dictated by the inpatient clinical care team. Following hospital discharge, titration of medication or, in the case of medication initiation, selection of the anti-hypertensive agent is based on clinical judgment from the call center physician. There are currently no standardized management guidelines for specific anti-hypertensive agents or parameters for medication titration in the postpartum period.^6^ Individuals with symptoms including chest pain, severe headache, blurry vision or vision disturbances, shortness of breath or blood pressures ≥ 180 mmHg systolic or 120 mmHg diastolic are referred to the emergency room (ER) for further evaluation.

### Outcomes

For the first objective, we compared demographic, clinical characteristics, inpatient and outpatient blood pressure and care utilization among three groups of postpartum individuals: (1) those who never required anti-hypertensive agents, (2) those initiated on anti-hypertensive agents during the delivery hospitalization prior to discharge and (3) those who were initiated on anti-hypertensive agents through the remote blood pressure management program after hospital discharge. Across group comparisons were performed using chi-squared for categorical data, one-way ANOVA for normally distributed data and Kruskall-Wallis for non-parametric data. Between group comparisons (comparing those requiring initiation of medications after hospital discharge to those never needing medications and to those who were discharged on medications) were performed using chi-squared for categorical data, two sample t-test for normally distributed data and Wilcoxon rank sum test for non-parametric data. We utilized logistic regression to model odds of ER visits and postpartum readmission within the UPMC Hospital system. Models were adjusted for predefined covariates known to be associated with blood pressure, postpartum ER visits and readmissions including maternal age, pre-pregnancy body mass index (BMI), self-reported race (as a social construct), type of hypertensive disorder, postpartum length of stay following delivery and delivery type. All postpartum hospital readmissions and ER visits within the UPMC Hospital System were captured, regardless of whether the ER visit or readmission were primarily related to hypertension given that there may be multiple reasons for hospital readmission or ER visits and our primary objective was to assess healthcare utilization in the postpartum period. We performed a sensitivity analysis limiting the analysis to hospitalizations that occurred after initiation of anti-hypertensive medications.

Additionally, we compared outpatient blood pressure trajectories in the first two weeks postpartum utilizing mixed-effects regression models with weeks postpartum as the timescale for all analyses. We used repeated blood pressure measures to fit mixed-effects linear regression models with each blood pressure measure as the outcome, participant identifier as random intercepts and weeks postpartum as a fixed effect expressed using restricted cubic splines with three knots positioned at 3, 8 and 13 days postpartum. The optimal number of knots were chosen comparing AIC and BIC between models. Mixed effects regression models were further adjusted for predefined covariates known to be associated with blood pressure including early pregnancy body mass index (BMI), maternal age and type of hypertensive disorder. Differences between the groups were tested via likelihood ratio test between models. There were 10% of individuals missing information on early pre-pregnancy BMI and missing data was handled with multiple imputation.

In our second objective, we interrogated the ability of the ACOG ≥ 150/100 mmHg BP threshold for medication initiation applied inpatient to identify individuals ultimately requiring anti-hypertensive agents. We categorized individuals with persistent postpartum inpatient systolic BP ≥ 150 mmHg or diastolic ≥ 100mmHg (2 or more BPs ≥ 4 hours apart) as “meeting ACOG criteria for medication initiation”. We compared the proportion of individuals who met ACOG criteria applied inpatient within each of the three groups. Finally, we assessed whether lower BP thresholds applied inpatient would more accurately identify (1) individuals ultimately requiring anti-hypertensive agents or (2) those who had severe hypertension (≥ 160/110 mmHg) after hospital discharge reported through our remote monitoring program. Given that the American College of Cardiology / American Heart Association (ACC/AHA) threshold for initiation of anti-hypertensive agents outside of pregnancy or postpartum in the absence of a known history of chronic hypertension is 140/90 mmHg, we utilized cutoffs between 140–150 mmHg systolic and 90–100mmHg diastolic in our analyses.

All analyses were performed using Stata IC 16 software package (StataCorp LP, College Station, TX). All p-values were 2-sided with a significance level of 0.05.

## Results

A total of 2,705 postpartum individuals were enrolled in the program and met inclusion criteria for this analysis between September 2019 and June 2021, mean age 29.8 ± 5.7 years, median early pregnancy BMI 28.1 [IQR 23.9–34.0] kg/m^2^. Most individuals in our cohort, 1,458 (53.9%), required no anti-hypertensive medications postpartum, 637 (23.5%) were discharged on anti-hypertensive medications, and 610 (22.6%) required initiation of anti-hypertensive medications after discharge. Among those requiring initiation of medications after hospital discharge, the median time of initiation of anti-hypertensive medication was postpartum day 7 [IQR 5–9]. We first compared demographic and clinical characteristics between individuals who never required anti-hypertensive agents to those requiring medication initiation after hospital discharge to determine whether clinical or demographic characteristics could differentiate the groups. When compared to those who never required anti-hypertensive medications, those who were initiated on medications after hospital discharge were older, had a higher early pregnancy BMI and were more likely to self-identify as Black race. The groups were similar in terms of mode of delivery, type of hypertensive disorder and length of stay postpartum. As expected, the group started on anti-hypertensives during the delivery hospitalization were more likely to have a diagnosis of preeclampsia, have a preterm delivery, lower infant birthweight, were more likely to receive magnesium and had a longer length of stay postpartum when compared to the other two groups (Table 1).

**Figure 1:**
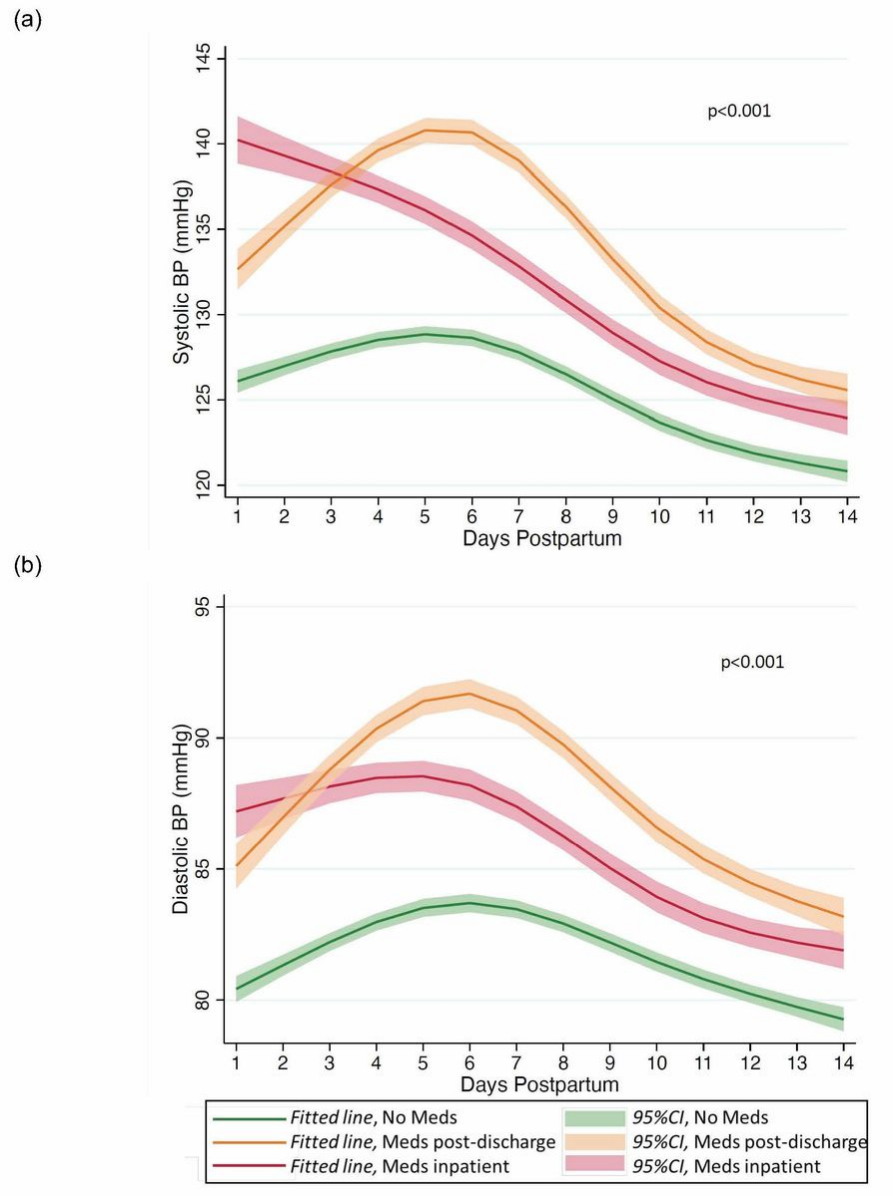
Fitted (a) systolic and (b) diastolic BP trajectory (solid line) and 95% CI (shaded area) in first two weeks postpartum by anti-hypertensive medication status; individuals requiring no anti-hypertensive medications (shown in green), individuals initiated on anti-hypertensive medications inpatient (shown in red), individuals initiated on anti-hypertensive medications post-discharge (shown in yellow).

We then compared inpatient and outpatient mean and maximum postpartum blood pressures among the three groups. During the inpatient postpartum hospitalization, individuals initiated on anti-hypertensive agents prior to discharge had the highest mean and maximum systolic and diastolic blood pressures (Table 2). However, when we compared outpatient postpartum blood pressures, individuals who required initiation of medications after hospital discharge had the highest mean and maximum systolic and diastolic blood pressure. Similarly, when we compared outpatient postpartum blood pressure trajectories between the three groups, we again found that the individuals who required initiation of anti-hypertensive medications after hospital discharge had the most adverse systolic (p < 0.001) and diastolic (p < 0.001) blood pressure trajectory with an exacerbation of hypertension between postpartum days 4 and 9 (Fig. 1). These differences in blood pressure trajectory across the groups persisted after adjustment for early pregnancy BMI, maternal age and type of hypertensive disorder. Overall, 382 (14%) individuals reported a blood pressure in the severe hypertension range after hospital discharge. Among all individuals with severe hypertension after hospital discharge, 180 (47%) were in the group started on medications after hospital discharge (Table 2).

We then compared care utilization postpartum among the three groups. When compared to individuals who never required anti-hypertensive agents and those initiated on medication while inpatient, individuals who required initiation of anti-hypertensive medication after hospital discharge were the most likely to have an ER visit (18.0% vs. 8.8% vs. 11.5%; p < 0.001) and a postpartum hospital readmission (12.1% vs.2.5% vs. 4.2%; p < 0.001) when compared with those who never required anti-hypertensive agents and, respectively. In adjusted analyses, compared to those who never required anti-hypertensive medications, individuals initiated on anti-hypertensive agents after hospital discharge had increased odds of an ER visit [aOR 2.22 (95%CI 1.65–2.98)] and increased odds of postpartum hospital readmission [aOR 5.73 (95%CI 3.72–8.82)] (Table 3). These results were similar when we limited our analyses to those who had medication initiated prior to hospital readmission (n = 56/74; 76% of readmissions; aOR 4.99 (95%CI3.16–7.88)).

For our second objective, we assessed the ability of the ACOG antihypertensive medication initiation guidelines, when applied to inpatient BPs, to identify individuals who (1) ultimately required anti-hypertensive agents and (2) had severe hypertension after hospital discharge. Of those who never required medications, 364 (25%) met the persistent 150/100 mmHg threshold inpatient, compared with 225 (37%) of those who were initiated on medications after hospital discharge and 478 (75%) of those who were treated with medications while inpatient (p < 0.001). Overall, the ACOG 150/100 mmHg threshold, when applied inpatient, identified 56% of individuals who ever needed medications and 51% of those who had severe hypertension after hospital discharge. In our cohort, using a threshold of 140/90 mmHg inpatient for initiation of anti-hypertensives would have identified 84% of those who ever required anti-hypertensive agents and 85% of those who had severe hypertension after hospital discharge but would have potentially overtreated 69% of those who never required medications (Table 2).

## Discussion

In our remote monitored population, over 20% of individuals required initiation of anti-hypertensive agents after discharge from the delivery hospitalization. Despite having clinical evidence of less severe disease at the time of delivery, individuals requiring initiation of anti-hypertensive medications after discharge were the most likely to have severe hypertension postpartum and had the highest rates of ER visits and hospital readmissions when compared to individuals who never required medications or those initiated on medications during the delivery hospitalization. These findings persisted when we limited our analysis to the hospital encounters that occurred after initiation of anti-hypertensive medication, suggesting that ER visit or hospital readmission was not the only factor driving initiation of medication. Further, current ACOG guidelines fail to identify over 60% of postpartum individuals who required medication initiation after discharge. However, lowering blood pressure thresholds for initiation of medication to align with guidelines in a non-pregnant or postpartum population may overtreat nearly 70% of individuals who never required anti-hypertensive agents postpartum.

Our findings demonstrate that individuals requiring medication initiation after hospital discharge have the highest rates of ER visits and hospital readmission even in the setting of a robust remote blood pressure management program, rates that may be even higher at institutions without such programs. These data support the critical role of remote BP monitoring and BP management programs in the postpartum period for detection of hypertension, particularly severe hypertension, after hospital discharge that requires anti-hypertensive medication initiation. Multiple studies have demonstrated the feasibility and acceptability of home blood pressure monitoring and management in the postpartum period.^13–15^ Prior small, randomized trials have shown a reduction in hospital readmission and increased adherence to ACOG recommendations surrounding BP assessment in the postpartum period.^13,16,17^ Even more notably, prior studies have also demonstrated a reduction in racial disparities in blood pressure measurement in the postpartum period.^18^ Despite this, few institutions have implemented system-level remote monitoring programs in the postpartum period. This is likely due to the significant barriers of implementing such programs, including technological barriers, lack of infrastructure, cost of the devices and lack of reimbursement.^19^ These cost-related barriers are likely exacerbated by the lack of insurance coverage in many states for telehealth and remote monitoring programs.^20^

Our findings regarding blood pressure trajectories in the immediate postpartum period are similar to those from smaller studies in this population, which suggest an exacerbation of hypertension after hospital discharge. These findings reinforce the importance of blood pressure monitoring after hospital discharge and consideration for lower thresholds for medication initiation in the postpartum period.^8,21^ For example, for individuals with blood pressures in the upper 140s systolic or upper 90s diastolic in the immediate postpartum period, providers should expect that exacerbation of hypertension after hospital discharge may result in blood pressures that fall in a range where ACOG would recommend medication initiation (≥ 150/100 mmHg) or even into the severe range (≥ 160/110 mmHg; approximately 30% in our cohort). This severe hypertension in the postpartum period has been associated with an increased risk of stroke, seizure and heart failure.^9,22^ Thus, our findings raise the question of whether a lower threshold for anti-hypertensive medication initiation ought to be considered in populations where home blood pressure monitoring and management is not utilized. On the contrary, universally employing a lower threshold, such as the 140/90 mmHg threshold that is now used for treatment of chronic hypertension in pregnancy based on the recently published Chronic Hypertension in Pregnancy (CHAP) trial may lead to overtreatment and potential adverse effects.^23^ While this is a widely accepted threshold for medication initiation outside of the postpartum period, obstetric guidelines use a higher threshold of 150/100 mmHg, possibly related to theoretical concerns regarding the risks of hypotension in the postpartum period. However, these guidelines were created prior to the use of remote monitoring and management of blood pressures after hospital discharge. The optimal threshold for initiation of anti-hypertensive medication initiation inpatient in the postpartum period is still unclear and future trials should address this important question. Our findings overall support the need for improved tools for risk stratification to predict which individuals may have significant worsening of hypertension postpartum. Despite the significant contribution of postpartum hypertension to severe maternal morbidity and hospital readmission, to date, few studies have developed risk prediction models or biomarkers specifically to predict which individuals might have worsening of hypertension after hospital discharge. Goel et al demonstrated that a high sFlt1/PlGF ratio was associated with an increased odds of persistent hypertension while inpatient postpartum, however, did not address blood pressure patterns after hospital discharge.^24^ Finally, it is worth noting that despite this study being performed in a large, tertiary care center with a delivery volume of 10,000 deliveries per year, over 20% of individuals in our study were not started on anti-hypertensive medications despite meeting ACOG criteria during the delivery hospitalization. This is certainly an area for future quality improvement.

Our study is strengthened by the large sample size and ability to compare risk factors across three different groups. Additionally, we used real-time home blood pressure measures instead of in-office measures that may be limited in this population. Our remote monitoring program incorporates standardized protocols, minimizing variation in practice. Our program has been robustly implemented at our institution, such that we have captured > 80% of the eligible population who delivered during the target time period. However, it is possible that our findings may be biased by not including those who did not participate in the program.^11^ Our study has limited generalizability given that our data are from a single institution and findings may not be the same among institutions without a remote blood pressure monitoring and management program. Furthermore, we are limited to evaluating postpartum care utilization only within our health system. All blood pressures are self-reported and we were unable to assess medication adherence during our study period. In our primary analysis, we assessed all ER visits and hospital readmissions regardless of the timing in relation to medication initiation, however, our findings were unchanged when we limited our analysis to hospital readmission after medication initiation, suggesting that it was not solely the interaction with the healthcare system that prompted initiation of anti-hypertensive medication. Additionally, we included all ER visits and rehospitalizations, not just those primarily related to hypertension in an effort to characterize care utilization for other reasons that may indirectly be related to hypertension (such as headache or heart failure). Given our limited sample size, we are unable to perform predictive modeling to assess risk factors and blood pressure thresholds for initiation of anti-hypertensive medications in the immediate postpartum period. Future study should attempt to identify the ideal thresholds for medication initiation postpartum, which may require a large-scale randomized controlled trial to address.

In summary, we found that nearly half of individuals treated with oral anti-hypertensive medication postpartum required initiation after discharge from the delivery hospitalization. In our cohort, individuals who required initiation of anti-hypertensive medications after discharge had the highest rates of ER visits and hospital readmission, despite clinical evidence of less severe disease during the delivery hospitalization. Importantly, current ACOG guidelines fail to identify the majority of individuals who require post-discharge medication initiation, reinforcing the critical role of remote BP monitoring in the postpartum period. Our findings also suggest the need for further research to develop effective blood pressure thresholds for anti-hypertensive medication initiation in the postpartum period.

## Figures and Tables

**Table 1.** Demographics and clinical characteristics of cohort (n=2,705) by anti-hypertensive medication status. p values reflect across group differences.

	No antihypertensive medications n=1458	Antihypertensive medications initiated inpatient n=637	Antihypertensive medications initiated post-discharge n=610	value
Age (years)	29.2 (5.6)*	30.7 (5.9)	30.5 (5.4)	<0.001
Early pregnancy BMI (kg/m^2^), median [IQR]	27.8 [23.7, 34.0]*	27.5 [23.9, 32.7]*	29.5 [24.8, 34.9]	<0.001
Self-identified race, n(%)				
White	1151 (78.9%)*	459 (72.1%)	438 (71.8%)	0.001
Black	226 (15.5%)	134 (21.0%)	128 (21.0%)	
Other	81 (5.6%)	44 (6.9%)	44 (7.2%)	
Insurance status, n(%)				0.55
Commercial	929 (63.7%)	394 (61.9%)	392 (64.3%)	
Public insurance	503 (34.5%)	238 (37.4%)	208 (34.1 %)	
Self-pay / Other	26 (1.8%)	5 (0.8%)	10 (1.6%)	
Primiparous, n(%)	752 (51.6%)	318 (49.9%)	308 (50.5%)	0.76
Gestational Diabetes, n(%)	144 (9.9%)	65 (10.2%)	77 (12.6%)	0.17
Birthweight (grams)	3174 (577)	2826 (810)	3144 (567)	<0.001
Cesarean Delivery, n(%)	530 (36.4%)	321 (50.4%)*	247 (40.5%)	<0.001
Type of hypertensive disorder, n(%)				
Preeclampsia	428 (29.4%)	391 (61.5%)*	203 (33.3%)	<0.001
Gestational hypertension	1030 (70.6%)	245 (38.5%)	406 (66.7%)	
Preterm delivery, n(%)	197 (13.5%)	233 (36.6%)*	88 (14.4%)	<0.001
Received magnesium, n(%)	221 (15.2%)*	356 (55.9%)*	118 (19.3%)	<0.001
Length of stay postpartum (days), median [IQR]	2.1 [1.7, 2.6]	3.0 [2.2, 4.1]*	2.1 [1.7, 2.7]	<0.001

Data are mean (SD) unless otherwise specified.

* Between group differences: Statistical significance (p value<0.05) for anti-hypertensive medications initiated after hospital discharge vs. each of the other categories denoted by

**Table 2.** Inpatient and outpatient blood pressures by anti-hypertensive medication status. p values reflect across group differences.

	No antihypertensive medications n=1458	Antihypertensive medications initiated inpatient n=637	Antihypertensive medications initiated postdischarge n=610	P value
Inpatient Postpartum Blood Pressure (mmHg)				
Mean Systolic	123 (9)*	131 (11)*	127 (9)	<0.001
Mean Diastolic	75 (7)*	81 (8)*	78 (7)	<0.001
Maximum Systolic	146 (16)*	161 (19)*	150 (15)	<0.001
Maximum Diastolic	97 (15)*	106 (15)*	100 (14)	<0.001
Outpatient Postpartum Blood Pressure (mmHg)				
Mean Systolic	123 (9)*	125 (9)*	128 (8)	<0.001
Mean Diastolic	80 (6)*	82 (6)*	84 (6)	<0.001
Maximum Systolic	138 (12)*	147 (13)*	152 (11)	<0.001
Maximum Diastolic	91 (8)*	97 (9)*	100 (8)	<0.001
Met ACOG criteria for antihypertensive medication initiation during delivery hospitalization (>150/100 mmHg), n(%)	364 (25%)*	478 (75%)*	225 (37%)	<0.001
Met ACC/AHA criteria for antihypertensive medication initiation during delivery hospitalization (>140/90 mmHg), n(%)	1002 (69%)*	564 (89%)*	516 (85%)	<0.001

Data are mean (SD) unless otherwise specified

* Between group differences: Statistical significance (p value<0.05) for anti-hypertensive medications initiated after hospital discharge vs. each of the other categories denoted by

**Table 3.** Clinical outcomes after hospital discharge by medication status.

	No antihypertensive medications n=1458	Antihypertensive medications initiated inpatient n=637	Antihypertensive medications initiated postdischarge n=610
Postpartum emergency room visit	128 (8.8%)	73 (11.5%)	110 (18.0%)
	Ref	aOR 1.22 (95%CI 0.86–1.74)	aOR 2.22 (95%CI 1.65–2.98)
Postpartum hospital readmission	36 (2.5%)	27 (4.2%)	74 (12.1%)
	Ref	aOR 1.85 (95%CI 1.04–3.30)	aOR 5.73 (3.72–8.82)
Severe hypertension after hospital discharge	79 (5.4%)	123 (19.3%)	180 (29.5%)
	Ref.	aOR 4.31 (95%CI 3.07–6.04)	aOR 7.12 (95%CI 5.25–9.67)

Data are n(%). aOR: adjusted odds ratio; adjusted for age, self-reported maternal race, early pregnancy BMI, postpartum length of hospital stay, delivery type

## Question

### Question:

How do outcomes compare among individuals with hypertensive disorders of pregnancy started on anti-hypertensive medication after hospital discharge to those who never require medications and those started on medications inpatient during the delivery hospitalization?

### Findings:

In this retrospective cohort study that included 2705 postpartum individuals enrolled in a remote blood pressure monitoring program, over 20% of individuals required initiation of anti-hypertensive medications after hospital discharge. These individuals had higher home blood pressures and increased odds of Emergency Room visits and hospital readmissions postpartum compared with individuals who never required anti-hypertensive medications and those discharged on anti-hypertensive medications.

### Meaning:

These data support the critical role of remote blood pressure monitoring programs and highlight the need for improved tools for risk strati cation and liberalization of thresholds for medication initiation postpartum.
